# Editorial: Brain stimulation: From basic research to clinical use

**DOI:** 10.3389/fnhum.2022.1092165

**Published:** 2022-11-28

**Authors:** Alia Benali, Ken-Ichiro Tsutsui, Masaki Sekino, Friederike Pfeiffer

**Affiliations:** ^1^Section Computational Sensomotorics, Hertie Institute for Clinical Brain Research and Centre for Integrative Neurocience, University of Tuebingen, Tuebingen, Germany; ^2^Laboratory of Systems Neuroscience, Graduate School of Life Sciences, Tohoku University, Sendai, Japan; ^3^Department of Bioengineering, School of Engineering, The University of Tokyo, Tokyo, Japan; ^4^Department of Neurophysiology, Institute of Physiology, Eberhard Karls University of Tuebingen, Tuebingen, Germany

**Keywords:** TMS (transcranial magnetic stimulation), rTMS (repetitive transcranial magnetic stimulation), tDCS, vagus nerve stimulation, noninvasive, neuromodulation

The aim of this Research Topic was to show how broad the field of brain stimulation has become recently, including basic research and clinical application. Numerous brain stimulation methods are being investigated to serve as neuromodulatory techniques, treating a variety of neuropsychiatric or neurological disorders (Antal et al., 2022; Camacho-Conde et al., 2022; Siebner et al., 2022). They can be divided into noninvasive and invasive methods. Non-invasive brain stimulation includes transcranial magnetic stimulation (TMS), transcranial direct current stimulation (tDCS) and transcranial alternating current stimulation (tACS), transcranial electrical stimulation (tES) and non-invasive vagus nerve stimulation (VNS). Invasive brain stimulation consists of intracortical microstimulation (ICMS) and deep brain stimulation (DBS).

Most of the original articles published here focused on brain stimulation using TMS. This section started with an overview article by Ueno, the inventor of the figure-of-eight coil, who, together with Masaki Sekino, provided an overview on figure-of-eight coils and their geometric variations. Figure-of-eight coils are advantageous for focal, localized stimulation, and are therefore already widely used in clinical applications. In addition, it is possible to achieve stimulation of deeper brain regions with different geometries of the coils. In general, a trade-off between depth and focality must be made. Tan et al. used a special double-cone coil to stimulate the cerebellar vermis with iTBS and study cerebral cortical excitability in healthy subjects exerting a balance task. The results showed, that even a single iTBS session increased HbO2 concentration measured with fNIRS, in the supplementary motor area but not in the dorsal lateral prefrontal cortex, although both structures are crucial during balance tasks. Mori et al. used a figure-of-eight coil to stimulate the superficially located primary motor cortex (M1). They investigated the differences in analgesic effects of applying rTMS to M1 to treat neuropathic pain. Results from three extracted clinical trials showed that rTMS was more effective in patients with neuropathic pain in the upper limb than in patients with neuropathic pain in the lower limb and face, thus the location of pain influences the analgesic effect of rTMS. Overall, TMS and various designs of figure-eight coils are a powerful tool to noninvasively investigate human neural networks. In daily clinical practice, attempts are made not only to stimulate focally or deeply with TMS, but also to shorten the duration of stimulation-time for the patient. This is more comfortable for the patient and economically favorable. One way to shorten the duration of stimulation was shown by the work of Matilainen et al., where active muscle contraction canceled the modulating effect of the interpulse intervals (IPIs) that are present in a resting muscle. The result suggested that IPIs as short as 2 s can be used to accelerate motor mapping with TMS in active muscles.

In general, most rTMS studies published here indicate that rTMS has a transient, short-lived effect on the network and that patient follow-up is necessary. Izuno et al. showed that the locally and transiently increased density of frontal sleep spindle activity in patients with major depressive episodes could be downregulated to baseline levels during the last half period of 10 consecutive rTMS sessions. The authors hypothesized that the increase was initially triggered by the high activity elicited by the high-frequency rTMS sessions and was then restored to baseline levels by an intrinsic homeostatic regulatory system between the cortex and thalamic loops. The clinico-cognitive correlation of this study with the rTMS-induced changes in sleep spindle density sheds light on the neuromodulatory effects of daily rTMS sessions on nocturnal sleep spindle activity.

A similar transient rTMS effect was observed by Fu et al., attempting to modulate network disorders in patients with refractory epilepsy with low-frequency rTMS over the vertex to achieve an antiepileptic effect. Almost all patients had visible motor seizures more than once per week during the ictal period. After stimulation, a positive result was obtained at the first follow-up, but it disappeared during the observation periods, indicating temporary antiepileptic efficacy for the patient and a necessity for sustained treatment.

Concerning rehabilitation, Brihmat et al. investigated 20 different rTMS protocols for their potential to improve functional outcomes after spinal cord injury. Preliminary results suggest benefits for motor and sensory recovery and are thought to be due to changes in corticospinal excitability and connectivity as well as cortical inhibition, altogether altering spinal circuits. Safety, tolerability and persistency need to be determined, and individually targeted rTMS interventions designed.

Could the rTMS therapy be improved in the long term by combining different brain stimulation techniques? Tang et al. demonstrated that electroacupuncture (EA) and TMS can modulate cerebral cortical excitability. Before EA, the right swallowing motor cortex of healthy subjects was more excitable by TMS than the left cortex at rest. After the EA, however, both cortices showed a modulation, with effects being more pronounced the right swallow motor cortex, and absent in the sham treatment group.

Geffen et al. investigated the effects of slow oscillatory (SO) tACS on motor cortical excitability with TMS, showing a significant increase in TMS-induced motor evoked potential amplitudes that persisted throughout the stimulation period. The increase was not simply due to anodal stimulation, because the acute effects of SO tACS were independent of phase and therefore do not support entrainment of endogenous slow oscillations as an underlying mechanism. These two studies showed that TMS can be combined with other brain stimulation methods, but the extent to which the combination prolongs the effects remains to be elucidated.

Importantly, not only positive effects and results supporting the hypothesis should be reported. The work of Rittweger et al. is a well-conceptualized paper, including the effects of sham treatment. In this study, rodent models of maternal immune activation (MIA) were used to answer the question: Is rTMS an alternative therapy for schizophrenia? MIA offspring are primarily impaired in behaviors requiring attentional decisions. iTBS reduced some behavioral deficits in MIA rats, but was not superior to sham stimulation, thus the handling itself had an effect. The authors describe small differences in processing experience between sham and MIA rats in the novel object recognition task but only when MIA rats were treated at juvenile age.

The second part of the publications dealed with non-invasive tES. Thereby, electrical currents are delivered to the brain with the aim of modulating neuronal activity. tDCS is widely used and applied to the left prefrontal cortex to elicit wide-ranging behavioral effects, including improved learning ability and vigilance. The neural mechanisms and repetitive stimulation have not yet been adequately explored. In their work, Sherwood et al., have investigated the effects of repetition and stimulation intensity of tDCS on cerebral perfusion [cerebral blood flow (CBF)]. Three groups (sham, tDCS of 1 or 2-mA) were stimulated and measured on three consecutive days. Resting CBF was quantified before and after stimulation using arterial spin-labeling MRI and then compared with the sham-condition. A distinct increase in CBF was only observed in the stimulation groups, depending on stimulation strength and number of pulses, suggesting that the neuronal effects of stimulation persisted for at least 24 h. Like rTMS, the tDCS-effects appear to be short-lived after a single stimulation but may produce a cumulative effect with repeated stimulation.

tDCS is a promising tool to improve and accelerate motor rehabilitation after stroke, but variability in clinical trials makes evidence-based clinical application difficult. One factor of its variability has been attributed to the unknown effects of stroke lesion conductance on stimulation intensity in targeted brain regions. Volume conductance models are promising tools for determining optimal stimulation settings, but the lesion volume is not considered in these models. Van der Cruijsen et al. proposed a method combining MRI, EEG, and transcranial stimulation to experimentally estimate the conductance of cortical stroke lesions. They developed and tested an algorithm to estimate the conductance of stroke lesions. This development will increase the accuracy of models of volume conductance of stroke patients and may lead to improved and more effective configurations of transcranial electrical stimulation for this group of patients.

Markovic et al., summarized 30 articles considering tDCS and rTMS to modulate fear memory and extinction. Fear memory and extinction are impaired in anxiety disorders, post-traumatic stress disorder and obsessive-compulsive disorders. Studies in either healthy humans or the clinical population as well as animal models addressing tDCS or rTMS as therapeutic approaches to treat these disorders are discussed. Pattern, timing, and location of the stimulation are important parameters to consider in future noninvasive therapies to treat these disorders.

Nooristani et al., discussed the parameters of tES that need to be defined before applying tES to patients with difficulties in hearing, such as electrode positioning and stimulation patterns, and their effect on specific aspects of hearing, such as temporal and spectral processing or binaural integration and speech comprehension.

Mitsutake et al., reviewed the feasibility of noninvasive M1 anodal transcranial direct current stimulation to improve gait performance in stroke patients. Nine studies using comparable stimulation parameters, patients and readouts were analyzed for their efficiency when combining tDCS with repetitive gait training vs. subsequent gait training after the application of tDCS. The authors concluded that simultaneous application of anodal tDCS during repetitive gait training seems to improve walking abilities more.

Has brain stimulation been discussed with regards to aging? Siegert et al., screened a database of published articles on tDCS and analyzed 16 studies for their reported effects on cognitive abilities, declarative and working memory, in older patients. Depending on the task that should be improved and the condition of the patient, the area of the brain as well as stimulation parameters should be carefully chosen. Anodal tDCS applied to the left cortical hemisphere is recommended to improve age-associated cognitive decline. In addition, combination with cognitive training seems promising.

A third method of brain stimulation is the cranial nerve stimulation. Cranial nerves transmit information from the environment or sensations directly to the brain, determining and modulating brain function (Adair et al., 2020). Invasive and noninvasive methods of electrical cranial nerve stimulation are already being used in clinical, behavioral, and cognitive areas. Compared to previous methods, cranial nerve stimulation is unique in that it allows axon pathway-specific manipulation of brain circuits, including thalamo-cortical networks. VNS is already approved in the EU and US for the treatment of drug-resistant epilepsy, cluster headache, and depressive disorders.

Von Wrede et al. investigated the effect of taVNS in focal and generalized types of epilepsy and a control group. The results showed that short-term taVNS affects the global characteristics of EEG-derived functional brain networks differently in the epilepsy groups than in the control group, and taVNS-induced changes in global network characteristics differed significantly between epilepsy types. No discernible spatial pattern was detected on the local network scale, indicating a rather nonspecific and generalized change in brain activity. The effects of such a nonpharmaceutical intervention need further investigation.

taVNS is unapproved as a treatment for stroke-associated dysphagia. After stroke, dysphagia may occur and is probably the most important factor for aspiration pneumonia, malnutrition, and dehydration. The vagus nerve, along with other cranial nerves, plays an important motor and sensory role in the regulation of swallowing. Long et al. investigated the effect of taVNS on cortical white matter and dysphagia symptoms in a rodent stroke model (cerebral ischemia). The results revealed that taVNS effectively improves dysphagia symptoms, increases remyelination, induces angiogenesis, and inhibits inflammatory response in the white matter.

Yu et al. reviewed the potential of closed-loop taVNS systems to influence and balance the central and peripheral nervous systems as well as the autonomic nervous system. taVNS is already applied in a disease-oriented way: motor-activated taVNS for upper limb rehabilitation, and respiratory-gated auricular vagal afferent nerve stimulation for pain/migraine patients. The authors further discussed the potential of additional future applications to treat neurological disorders, cardiovascular diseases, or diabetes.

Unlike noninvasive brain stimulation methods, invasive stimulation methods are more focal but require neurosurgery. Li et al. addressed the prevention and treatment of hardware-related infections during DBS surgery. The authors showed that the intraoperative irrigation with hydrogen dioxide solution during the implantation of the implantable pulse generator (IPG) reduced the incidence of primary infections. For late-infections (after 3 months), the authors developed a strategy called Isa that helps to prevent the occurrence of secondary IPG infections.

The other two publications focused on ICMS in humans and rats.

Investigations on humans are not ethically approved and can only be performed with restrictions. In the present case study by Long et al., intracranial electrodes were implanted in a 30-year-old female patient to localize the source of drug-resistant seizures. The results of the direct cortical stimulations were recorded with a stereo-EEG. The authors were able to elicit two types of auditory hallucinations and confirmed hierarchical processing of auditory information in humans.

The goal of cortical neuroprosthetics is to feed sensory information directly into the cortical network as precisely as possible. However, sensory processing is dependent on behavioral context. Therefore, a particular behavioral context may alter stimulation effects and thus perception. In the study by Butovas and Schwarz, rats were operantly conditioned to move a whisker in a target-specific manner. The authors were able to investigate the effect of a passive or active touch (the absence or presence of a whisker movement) at the cortical level and gain a deeper understanding of the underlying mechanism.

Finally, Liu et al. summarized recent advances in adjusting second-generation brain stimulation techniques that aim at neuromodulation in humans. Noninvasive focused ultrasound did not only alter neuronal activity and influenced behavior but was also shown to cause responses at the molecular level. Temporal interference stimulation can reach deep brain regions noninvasively and is feasible to precisely regulate subcortical structures, influence several cell types and is a promising future alternative for deep brain stimulation. Near-infrared optogenetic stimulation requires the insertion of responsive nanoparticles in the target cells that respond to the near-infrared light. Thus, it is minimally invasive but offers the possibility to activate specific cells. These approaches would provide the possibility to modulate neural activity in a more targeted way, but still need a lot of investigation before application in humans would be possible.

Overall, noninvasive methods of brain stimulation are already used as therapy for certain neurological disorders but hold promise for many additional and more specific applications in the future. Researchers are working hard to improve the techniques, refine the results, and understand the underlying mechanisms.

## Author contributions

All authors listed have made a substantial, direct, and intellectual contribution to the work and approved it for publication.

## Funding

This work was supported by DFG, Germany, Grant No. BE 6048/2-1 (to AB). FP has received funding from the European Union's Horizon 2020 research and innovation program under the Marie Sklodowska-Curie grant agreement No. 845336 NG2-cells.

## Conflict of interest

The authors declare that the research was conducted in the absence of any commercial or financial relationships that could be construed as a potential conflict of interest.

## Publisher's note

All claims expressed in this article are solely those of the authors and do not necessarily represent those of their affiliated organizations, or those of the publisher, the editors and the reviewers. Any product that may be evaluated in this article, or claim that may be made by its manufacturer, is not guaranteed or endorsed by the publisher.
